# Kin count: Social, physiological, and environmental correlates of annual survival in Belding’s ground squirrels

**DOI:** 10.1371/journal.pone.0358786

**Published:** 2026-11-04

**Authors:** Jill M. Mateo, Katherine C. Brooks, Andy Dosmann

**Affiliations:** 1 Department of Comparative Human Development, The University of Chicago, Chicago, Illinois, United States of America; 2 Committee on Evolutionary Biology, The University of Chicago, Chicago, Illinois, United States of America; Northern Michigan University, UNITED STATES OF AMERICA

## Abstract

Long-term studies of free‑living animals provide opportunities to identify the traits and environmental conditions that predict survival under natural selection. Using over two decades of mark-recapture demographic data, we evaluated intrinsic, social and environmental predictors of annual survival in a population of Belding’s ground squirrels (*Urocitellus beldingi*), obligate hibernators that exhibit female philopatry and kin-structured social groups. We assessed the effects of age, sex, body mass, glucocorticoid physiology, reproductive effort, kinship and snowfall using sex-specific generalized linear mixed models and logistic regressions. Adult females had higher survival than adult males, whereas juveniles—particularly males—experienced the highest mortality. As expected, survival was strongly associated with body condition: heavier individuals and those with lower fecal glucocorticoid metabolites were more likely to survive. For the first time, we demonstrate that the number of relatives predicted survival in this species. That is, adult females’ probability of surviving to the next summer increased with the specific number of living female close kin, beyond the effect of kin presence alone. Male survival was unrelated to kinship. In contrast, juvenile females with more kin had lower survival, suggesting age‑specific trade‑offs associated with kin structure. Earlier emergence from hibernation improved juvenile survival and was also associated with successful adult female reproduction. Snowfall influenced survival through phenological pathways: lower snow before the active season was associated with earlier emergence and higher survival in adult females, while post‑season snow was not related to survival. Together, these results show that social structure, physiological state, and climate‑driven seasonal timing interact to shape annual survival in an obligate hibernator, with effects that vary by sex and life stage. Understanding these interactions will be increasingly important as snowfall declines across much of *U. beldingi*’s range.

## Introduction

For most animals, the extent to which reproduction and survival are influenced by kinship systems remains incompletely understood. In some taxa, including primates and ground squirrels, the presence of adult kin is associated with enhanced reproductive success or survival. For other sciurids, proximity to kin can have mixed effects [1–3]. Using a demographic database spanning more than two decades, we examined whether kinship influences annual survival in wild Belding’s ground squirrels (*Urocitellus beldingi*), while accounting for intrinsic and extrinsic factors arising from their life history, physiology and social system.

Belding’s ground squirrels exhibit strong nepotistic behaviors. Females with living close kin (mothers, daughters, and sisters) are more likely to produce risky alarm calls and defend territories of related females against potentially infanticidal conspecifics. In contrast, males disperse from their natal areas, rarely live near kin and do not exhibit nepotistic behaviors [4]. In mammals, social organization can influence survival and longevity when kin‑biased cooperation alters exposure to ecological challenges or physiological stressors [5]. This pronounced sex difference in dispersal and social behavior makes *U. beldingi* an especially tractable system for testing whether annual survival is influenced by the number of living kin in a sex‑ and age‑dependent manner.

Kin effects on survival cannot be evaluated in isolation, particularly for obligate hibernators. In *U. beldingi*, annual survival is likely influenced by body condition, physiological state, age and environmental conditions [e.g., 6–8]. Long‑term data from free‑living populations allow these species-relevant traits to be assessed in their natural context, capturing temporal variation in both biotic and abiotic drivers of survival.

Energetically demanding life‑history stages, including migration or hibernation, require sufficient energy reserves. For hibernators that do not cache food, accumulation of body mass before winter is critical. Many ground‑squirrel species are vulnerable to starvation or freezing during torpor and must balance time spent foraging with antipredator vigilance during the active season. Consequently, the relationship between pre‑hibernation body mass and annual survival varies with sex, age and reproductive history [e.g., 9–11].

Body mass and energy balance are in turn regulated by glucocorticoids, which mediate energy mobilization and storage and coordinate physiological responses to unpredictable or uncontrollable stressors [12,13]. These hormones elevate circulating glucose, enhance cardiovascular tone and suppress non‑essential processes. Across vertebrates, including sciurids, relationships between baseline glucocorticoids and subsequent survival are mixed. However, chronic or repeated activation of the hypothalamic–pituitary–adrenal axis may impair health and reduce lifespan, particularly in harsh or variable environments [6,12–15].

Reproductive investment may also shape survival. Although theory predicts a trade‑off between current reproduction and future survival, empirical patterns remain inconsistent, even within taxa [5,16]. Repeated measurements from known individuals therefore provide an opportunity to test whether reproductive effort influences annual survival in male and female *U. beldingi*, while controlling for condition and environmental variation.

In addition to intrinsic traits, ecological conditions strongly affect survival in alpine and montane species. Animals reproducing at high elevations typically migrate downslope or hibernate in place to survive winter. Snowfall may enhance survival by insulating hibernacula, whereas delayed snowmelt or late spring storms can lower survival or subsequent reproductive output [8,10,17]. Although the study population examined here has persisted long‑term, approximately 42% of *U. beldingi* populations have disappeared over the past century, likely due to declining snowfall [18]. Long‑term datasets are therefore essential for understanding the effects of climatic variation on survival in this species.

Variation in survival arises from interactions among intrinsic and extrinsic factors that shape population demography and social structure. These factors both influence and are influenced by sex‑biased dispersal, operational sex ratios and mating strategies, ultimately determining mortality patterns and the targets of natural selection [5]. Here, we first describe demographic characteristics of our study population, including population size, sex ratio and longevity. We then evaluate how intrinsic factors (age, sex, body mass, glucocorticoids and reproductive status) and extrinsic factors (snowfall and number of living female close kin) contribute to annual survival. Based on previous research on ground‑dwelling squirrels [4,7,10,12,19], we expected body mass and total snowfall to be positively associated with annual survival, whereas glucocorticoid levels would be negatively associated. We also anticipated that any effect of close kin on survival would differ between the sexes, given their contrasting dispersal patterns, social behaviors and life‑history constraints. We made no a priori predictions for the remaining variables.

## Materials and methods

### Study system and field site

Belding’s ground squirrels are diurnal, obligate hibernators that inhabit alpine and subalpine meadows. Females are philopatric and form kin groups, whereas males disperse from their natal areas and do not associate with kin. We studied a free‑living population in Rock Creek Canyon, California (elevation 2835 m), from 1993–2017 (excluding 1995), and focused analyses on 1996–2012, when trapping effort was most consistent. Individuals were permanently marked with PIT or ear tags and monitored during the active season (May–July). Additional details on *U. beldingi* life history and the field site are provided in Mateo [20]. All procedures followed the Guide for the Care and Use of Laboratory Animals (National Institutes of Health) and the Guidelines of the American Society of Mammalogists for the use of wild mammals in research and education. Protocols were approved by the Institutional Animal Care and Use Committees at Cornell University (#00–32), the University of Chicago (# 71255) and the University of California – Santa Barbara (#532). Research was conducted under permits from California Fish & Wildlife (formerly Fish & Game) and the United States Forest Service.

### Demography and life-history data

From 1996–2012 we marked 1507 females and 1652 males, along with five individuals whose sex was not determined. Most ground squirrels were first captured as juveniles; animals first captured as adults were conservatively assigned an age of 2.5 years, an approach that introduces minimal and consistent error across years [e.g., 21]. Annual survival was defined as an individual’s presence in the population the following year.

### Covariates of survival

We evaluated intrinsic and extrinsic predictors of annual survival to the subsequent year, including age (2004–2012), body weight (2003–2012), fecal glucocorticoid metabolites (‘FGMs’; adults: 2005–2012; juveniles: 2002–2012), number of living adult female kin (2005–2012) and snow (1996–2012). The number of years in each analysis varied across covariates depending on the availability of data. Female reproductive effort was measured as production of a litter that survived to emergence aboveground (“weaners”), and litter size for weaned litters. Male reproductive effort was measured as the number of observed copulations during years with systematic behavioral observation during mating. Body weight and FGMs were measured repeatedly during the active seasons (*n* = 1–13 measurements of each individual each season); because there were no significant differences in survival across mating, birth and postnatal emergence periods as a function of these two variables, annual means were used in analyses. The number of living female close kin (mother, daughter or sister; range 0–7 for both sexes) was determined using maternal pedigree data for years with reliable records. Snowfall data (snow water equivalent, SWE in millimeters) were obtained from the nearest California Department of Water Resources daily snow sensor (‘RCK’, approximately 2000 m from the site at 2956 m elevation) and summarized as maximum snowfall for the winter preceding each active season (“snow‑pre”, examining survival as a function of preceding snowfall) or the winter following it (“snow‑post”, examining survival as a function of subsequent snowfall). Note that snow-post for active season x is the same value as snow-pre for active season x + 1. As a proxy for annual dates of emergence from hibernation, we backdated by 50 days (the duration of gestation and lactation) the date of natal emergence of each season’s first litter. By the time the first female in the meadow mates, all individuals have emerged from hibernacula (unpubl. data), and thus that date serves as a good proxy for the start of annual activity. Analyses were conducted separately for males and females because of sex differences in survival and life history. Juveniles were analyzed separately from yearlings and adults due to large differences in survival (see below). Juvenile analyses included body weight at natal emergence, emergence date, FGMs, maternal kin number, and snow‑post. Detailed field protocols and FGM procedures are provided in Mateo [20].

### Analyses

Survival of yearlings and adults was analyzed separately for males and females using generalized linear mixed models with binomial error distributions and logit links. Individual identity was included as a random effect to account for repeated measures. Juvenile survival was analyzed using sex‑specific logistic regression models. Predictor effects are reported as coefficients, test statistics, and odds ratios when applicable. Analyses were conducted in R using lme4 package; supplementary tests were performed in Systat. Sample sizes are provided in S1 and S2 Tables. Statistical significance was defined as *p* < 0.05.

## Results

### Demography and life-history data

Annual population size at Rock Creek Canyon, CA, ranged from a low of 42 in 1996 to a high of 497 in 2009. For animals that survived their first winter, maximum lifespan reached 9 years for both sexes with females living an average of 3.83 years and males 3.43 years. Sex differences in survival produced pronounced asymmetries in age structure (Fig 1). Across 365 litters, mean litter size was 4.93 ± 0.08 (range 1–9), with a male:female sex ratio of 1.02:1. Among yearlings and adults, sex ratios ranged from 0.29:1–0.84:1, indicating greater male mortality across ages.

**Fig 1:**
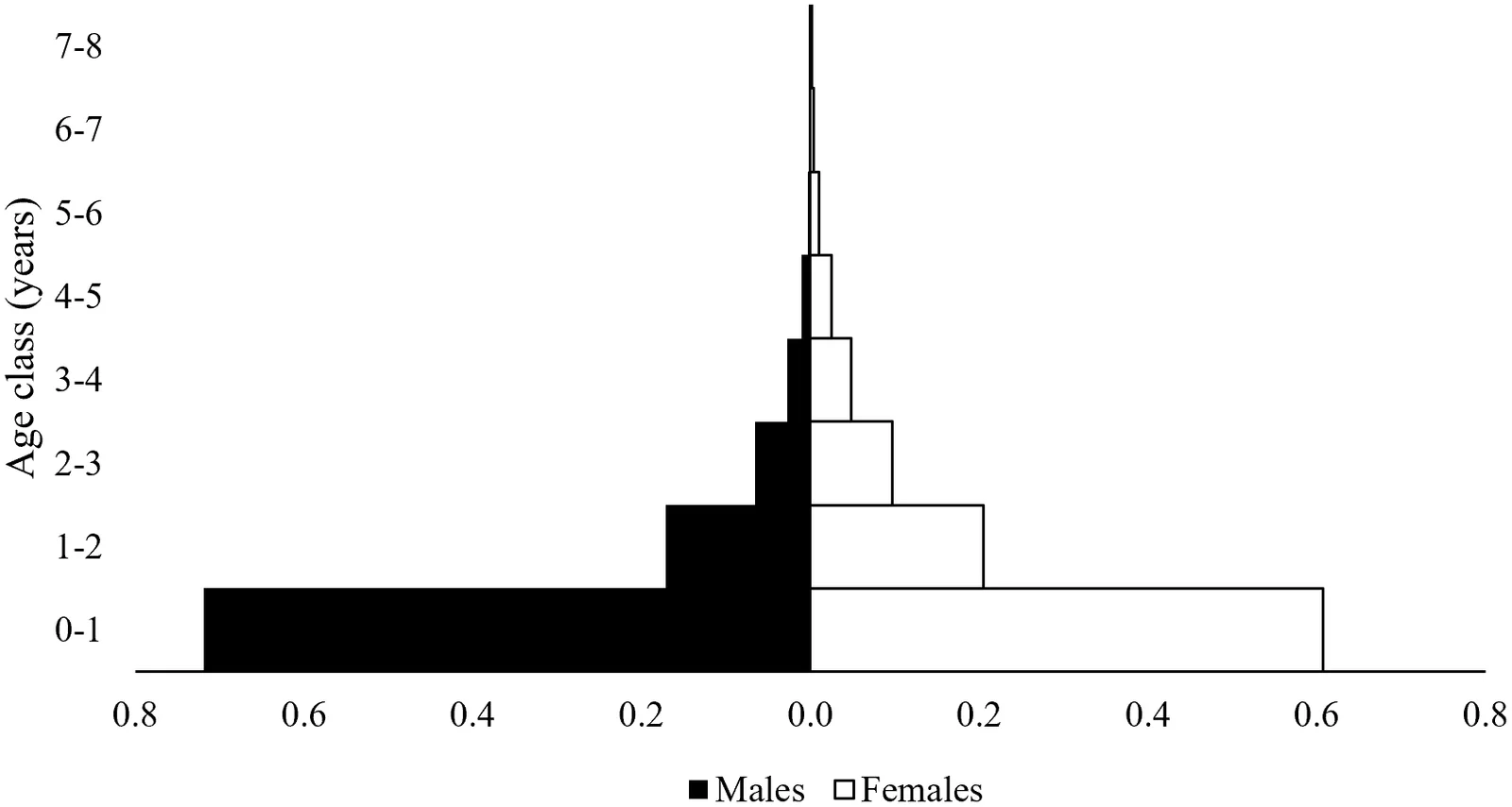
Age and sex structure of a population of Belding’s ground squirrels. Population pyramid showing age and sex composition of *Urocitellus beldingi* at Rock Creek Canyon, CA, from 1996–2011. Data capture the full lifespans of known-age juveniles and yearlings (*N* = 1784 males, 1948 females).

### Correlates of survival

Among ground squirrels of known age that survived their first hibernation, there was no significant difference in survival between yearlings and adults (283/645 and 236/505, respectively; *X*^2^ = 0.93, *p* = 0.33). Body weight was positively associated with survival for both sexes (Tables 1, 2; Fig 2); heavier individuals were more likely to survive to the following summer. In general, the existence of close kin was associated with higher survival in females (147/283 with kin versus 145/360 with no kin; *X*^2^ = 8.70, *p* = 0.003), but not in males (*X*^2^ = 0.03, *p* = 0.85). Focusing on the specific number rather than presence of kin, female survival increased with the number of living female relatives, whereas male survival showed no association. FGM levels were negatively associated with survival in females but not in males. Age did not predict survival for either sex. At the population level, snow in the winter preceding the active season (snow-pre) significantly predicted female survival; lower snowfall was associated with increased survival. Snow-pre did not predict male survival, and snow following the active season (snow-post) was not associated with survival in either sex (Tables 1, 2; Fig 2).

**Table 1:** Physiological, social and environmental predictors of survival in yearling and adult female *Urocitellus beldingi*. Results are from generalized linear mixed models with binomial error distributions and logit link functions.

Variable	Coefficient	Standard error	z	*p*
**Weight**	**0.010**	**0.002**	**4.709**	<0.001***
**Close kin**	**0.176**	**0.063**	**2.803**	0.005**
**FGM**	**−0.003**	**0.001**	**−2.612**	0.009**
Age	−0.127	0.093	−1.364	0.173
**Snow (pre)**	**−0.001**	**<0.001**	**−3.361**	<0.001***
Snow (post)	<0.001	<0.001	0.108	0.914

**Table 2:** Physiological, social and environmental predictors of survival in yearling and adult male *Urocitellus beldingi*. Results are from generalized linear mixed models with binomial error distributions and logit link functions.

Variable	Coefficient	Standard error	z	*p*
**Weight**	**0.009**	**0.002**	**4.191**	<0.001***
Close kin	0.054	0.083	0.651	0.515
FGM	−0.001	0.001	−1.106	0.269
Age	0.021	0.077	0.278	0.781
Snow (pre)	−0.001	<0.001	−1.351	0.177
Snow (post)	<0.001	<0.001	0.132	0.895

**Fig 2:**
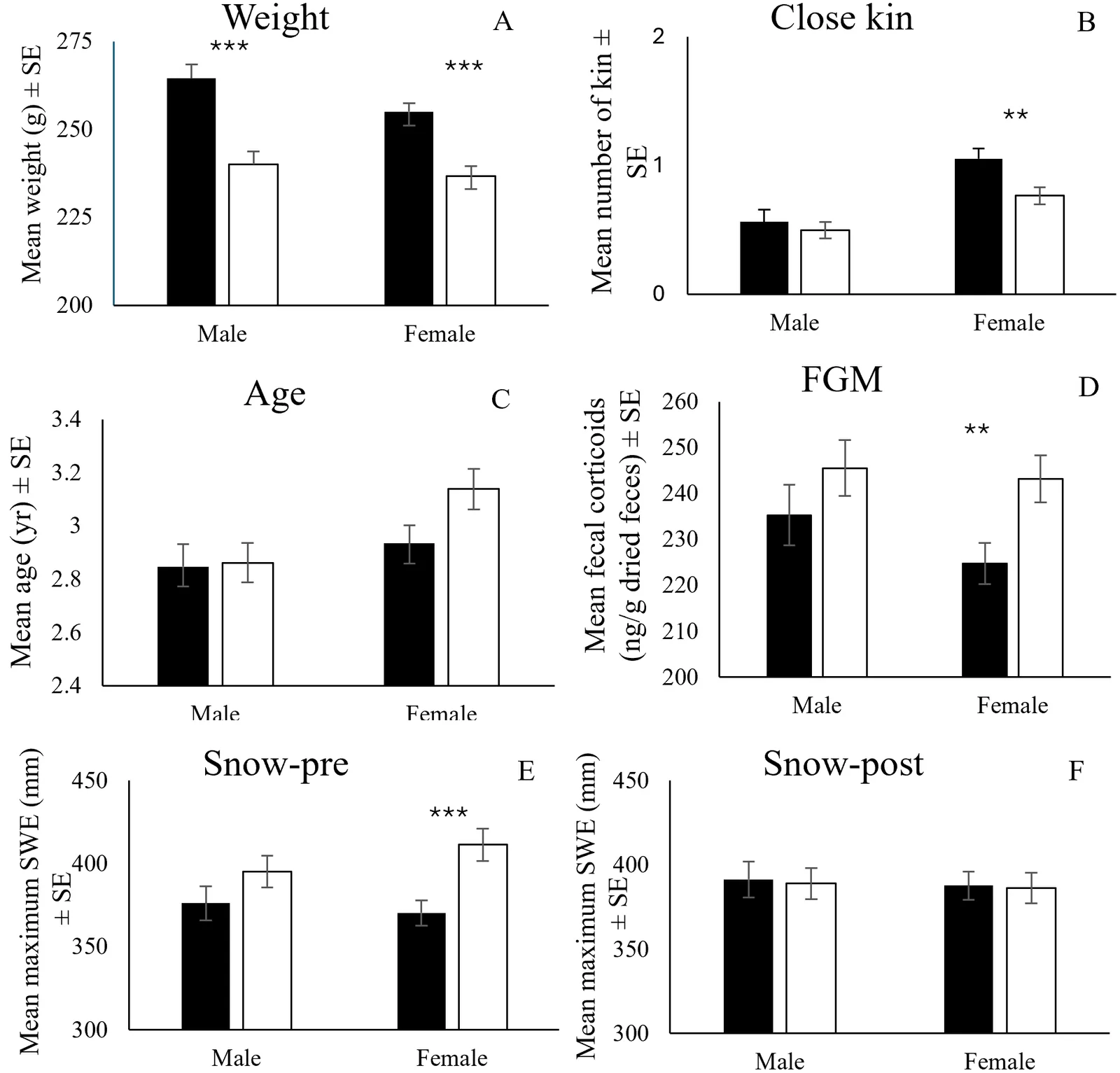
Correlates of annual survival in adult Belding’s ground squirrels. Potential correlates of survival by *U. beldingi* in Rock Creek Canyon, CA, showing mean and standard error of traits of adult males and females that survived to the following year (black bars) or did not (open bars). A) Individual body weight averaged over each summer. B) Number of living adult female close kin (*r* ≥ 0.25). C) Age in years. D) Fecal glucocorticoid metabolites averaged for each individual across each active season. E) Maximum snow during the winter prior to the active season (snow-pre), expressed snow water equivalent (SWE). F) Maximum snow during the winter following the active season (snow-post). Data are illustrative; statistical results are from generalized linear mixed models controlling for repeated measures (Tables 1, 2). **p < 0.01, ***p < 0.001.

### Reproductive effort and survival

Females producing litters that emerged aboveground were more likely to survive to the next year than females that did not (*X*^2^ = 28.876, *p* < 0.0001; weaners: 270/453; non-weaners: 144/355). Male survival did not differ between individuals observed copulating and those not observed copulating (*X*^2^ = 0.137, *p* = 0.711). Among weaning females, survival was unrelated to litter size (logistic regression: *n* = 453, *t* = 0.283, *p* = 0.777). Earlier emergence from hibernation was associated with successful rearing of litters by females (weaners versus non-weaners; *n* = 808, *t* = −5.886, *p* < 0.0001, odds ratio = 0.951 (0.935, 0.967)), but not copulation by males (*n* = 313, *t* = −0.824, *p* = 0.410). Females were also more likely to rear litters following winters with lower snow-pre (*n* = 808; *t* = −2.224, *p* = 0.026, odds ratio = 0.999 (0.998, 1.00)). Snow did not predict whether males copulated the following summer (*n* = 313, *t* = −1.874, *p* = 0.061).

### Juvenile survival

Female juveniles were more likely to survive to yearling age than male juveniles (263/1246 and 162/1335, respectively; *X*^2^ = 37.72, *p* < 0.0001). Survival was unrelated to body weight at natal emergence for either sex. However, earlier emergence dates were associated with higher survival for both sexes (Table 3). In general, female juveniles with female close kin alive during the summer had lower survival than those without kin (71/399 and 92/306, respectively, *X*^2^ = 14.67, *p* < 0.0001) whereas kin presence did not affect survival of juvenile males (*X*^2^ = 0.92, *p* = 0.337). When considering total kin number, young females with *more* living adult female kin had significantly lower survival than those with fewer relatives (*X̅=*1.26 kin for non-survivors and 0.92 kin for survivors, range 0–7; Table 3). Lower FGMs were associated with higher juvenile survival for both sexes. Snow-post did not predict juvenile survival.

**Table 3:** Predictors of juvenile survival to the yearling year in *Urocitellus beldingi*. Results are from sex‑specific logistic regression models.

Variable	Sex	*t*	*p*	Odds Ratio (95% CI)
Weight	Female	1.748	0.08	
	Male	−0.168	0.867	
Close kin	**Female**	**−2.411**	**0.016**	**0.846 (0.738, 0.969)**
	Male	−1.365	0.172	
FGM	**Female**	**−2.158**	**0.031**	**0.9965 (0.9932, 0.9997)**
	**Male**	**−2.045**	**0.041**	**0.9966 (0.9934, 0.9999)**
Emergence date	**Female**	**−4.782**	**<0.0001**	**0.957 (0.939, 0.974)**
	**Male**	**−2.485**	**0.013**	**0.974 (0.953, 0.994)**
Snow (post)	Female	−0.546	0.585	
	Male	−1.154	0.249	

## Discussion

We conducted a mark-recapture analysis using over two decades of individual‑based demographic data on a natural population of Belding’s ground squirrels. Our study demonstrates how social, physiological and environmental factors jointly shape survival in this obligate hibernator. As expected, survival was strongly associated with body condition and glucocorticoid physiology. Critically, we also show for the first time that survival—particularly for adult females—was positively associated not only with presence of close matrilineal kin, but with the specific *number* of such relatives. Together with the effects of climate‑driven phenological variation, these findings suggest that social structure mediates how ecological and physiological constraints translate into survival in a highly seasonal environment.

Consistent with patterns observed in other sciurids [8,22,23], juvenile *U. beldingi* —especially males— experienced lower survival than adults. Intensive trapping across the primary site and adjacent meadows suggests that reduced male detection in the yearling year reflected true mortality rather than dispersal. Among yearlings and adults, male mortality exceeded female mortality, generating pronounced sex asymmetries in age structure (Fig 1). These patterns likely reflect sex‑specific life‑history strategies: males incur substantial energetic and injury-related costs during the mating season, whereas females allocate resources to reproduction and subsequent mass gain for hibernation [4,24].

A central finding of this study is that social environment predicted survival in females. Prior studies in some mammals have shown an effect of kin presence on survival or fitness [e.g., 1, 2], including sciurids [e.g., 3, 25; cf. 26, 27]. Here, females with female close kin present in the meadow had higher survival than those without kin. Our dataset further reveals for the first time that the precise number of kin affected survival. Females with more adult female relatives were more likely to survive than those with fewer kin, whereas male survival showed no such relationship (Tables 1, 2; Fig 2). This sex difference is consistent with the philopatric nature of females, the dispersal of males and this species’ documented nepotistic behaviors [4]. The cumulative effect of kin number suggests additive or compounding advantages of kin-structured social environments. Males likely did not benefit because natal dispersal removes them from kin clusters. It is also possible that the sexes inhabit environments with differing qualities. For example, *U. beldingi* living at or reared near meadow edges tend to be more vigilant and have lower FGMs than those in the center [20]. Future work is needed to identify the specific behavioral or ecological pathways that link specific kin number to female survival. In contrast to adults, juvenile females with more living adult female kin had lower survival to yearling age (Table 3). Each additional close relative reduced the odds of survival by approximately 15%. This negative association likely reflects localized competition for resources such as food or refuge space among related females in a philopatric system [28]. These life‑stage differences suggest that kinship generates age‑specific trade‑offs: kin numbers impose costs in early development but yield benefits later in life.

Several mechanisms may underlie the positive effects of kin number on adult female survival. Close kin may reduce the individual burden of vigilance or territorial defense through cooperative alarm calling and defense of shared areas. In a related species, females near kin allocate more time to foraging and fewer resources to vigilance or competition [29]. Kin-based associations may also buffer individuals physiologically; social affiliation can reduce glucocorticoid levels and enhance immune function [13]. These behavioral and physiological pathways provide plausible mechanisms linking kin number to adult survival in this population.

As expected for an obligate hibernator that does not cache food, body mass strongly predicted survival. Heavier individuals of both sexes were more likely to survive from summer to summer, consistent with findings in other ground squirrels [e.g., 11, 12, 30]. In juveniles, emergence mass was not predictive of survival, but earlier emergence provided a substantial advantage by increasing the time available for growth and fat deposition prior to hibernation, highlighting the importance of seasonal timing during early life [e.g., 9].

Glucocorticoid physiology further influenced survival. Lower fecal glucocorticoid metabolites were associated with higher survival in adult females and in juveniles of both sexes (Fig 2D; Tables 1, 3), indicating that chronic or elevated stress responses may interfere with the accumulation of energetic reserves necessary for hibernation [see also 20]. The lack of a detectable effect in adult males may reflect their longer period for mass gain following the mating season compared with females [e.g., 8, 24].

Reproductive effort was positively associated with survival in adult females: individuals that successfully reared litters were more likely to survive than non‑reproductive females. Litter size did not predict survival. This pattern suggests that reproduction did not impose detectable survival costs [16,19,31]. Instead, it is likely that females that emerged earlier and experienced favorable environmental conditions were more likely both to reproduce successfully and to survive, indicating that timing and condition jointly mediate these outcomes.

Environmental conditions appeared to shape survival primarily through phenology rather than overwinter insulation. Snowfall after the active season did not predict survival, providing no support for the hypothesis that deeper snow enhances survival via insulation [17]. In contrast, lower snow prior to the active season was associated with higher survival in adult females (Tables 1–3). Using our proxy for the onset of seasonal activity, we found that reduced snow corresponded with earlier spring emergence, which may extend the foraging period necessary for mass gain. These effects were most pronounced in females, whose somatic investment to prepare for hibernation may be constrained by lactation [24].

Our findings suggest that survival in Belding’s ground squirrels is shaped by interactions among social structure, physiological state and climate‑driven seasonal timing, with effects that vary by sex and life stage. Adult female survival was enhanced by greater body mass, lower glucocorticoid levels, earlier emergence, and favorable social environments characterized by higher numbers of close kin. Importantly, the *number* of female close kin —rather than their mere presence alone— predicted survival, highlighting the significance of kin-structured social organization in this species and paralleling broader evidence that strong social bonds enhance longevity in mammals, particularly females [5]. As snowfall continues to decline across western North America [32], the interplay between social environment, physiological condition and changing climate phenology will be critical for predicting population persistence in obligate hibernators. Long‑term, individual‑based datasets such as this one provide essential insight into how multiple factors converge to shape survival in species inhabiting highly seasonal environments.

## Supporting information

S1 Table Sample sizes and numbers of unique individuals used in analyses of predictors of yearling and adult survival. (PDF)

S2 Table Sample sizes for analyses of juvenile survival to the yearling year. (PDF)
