# Supplementary material for: Kin count: Social, physiological, and environmental correlates of annual survival in Belding’s ground squirrels
Source: PLoS One. 2026 Nov 4;21(11):e0358786. doi: 10.1371/journal.pone.0358786 (PMC13734506; doi:10.1371/journal.pone.0358786)
Supplement: Supplementary file 1 [file pone.0358786.s001.pdf]

**Variable**	**Sex**	**Unique individuals**	***N***
Weight	Female	301	527
	Male	234	357
Close kin	Female	380	643
	Male	305	470
FGM	Female	305	508
	Male	237	356
Snow	Female	496	1016
	Male	426	719
Age	Female	344	608
	Male	275	438
