# Supplementary material for: Kin count: Social, physiological, and environmental correlates of annual survival in Belding’s ground squirrels
Source: PLoS One. 2026 Nov 4;21(11):e0358786. doi: 10.1371/journal.pone.0358786 (PMC13734506; doi:10.1371/journal.pone.0358786)
Supplement: Supplementary file 2 [file pone.0358786.s002.pdf]

**Variable**	**Sex**	***N***
Weight	Female	949
	Male	1033
Close kin	Female	783
	Male	841
FGM	Female	270
	Male	317
Emergence date	Female	1135
	Male	1221
Snow	Female	1246
	Male	1335
